# Tuberculosis after TB Preventive Therapy in Persons Living with HIV Recently Initiating Antiretroviral Therapy, Mozambique

**DOI:** 10.3201/eid3203.251349

**Published:** 2026-03

**Authors:** Lindsay Templin, Yagna Varajidas, Durval Respeito, Pereira Zindoga, Debora Weiss, Alexandre Nguimfack, Eudoxia Filipe, Arlete Mahumane, Maria Inês Tomo de Deus, José Mizela, Sonia Chilundo, Bruno Simoes, Aleny Couto, Ishani Pathmanathan, Emilio Dirlikov, Benedita José

**Affiliations:** US Centers for Disease Control and Prevention, Maputo, Mozambique (L. Templin, Y. Varajidas, D. Respeito, A. Nguimfack, A. Mahumane, M. Tomo de Deus, J. Mizela, S. Chilundo, I. Pathmanathan, E. Dirlikov); US Agency for International Development, Maputo (P. Zindoga); Ministry of Health Mozambique, Maputo (E. Filipe, B. Simoes, A. Couto, B. José); US Centers for Disease Control and Prevention, Atlanta, Georgia, USA (D. Weiss)

**Keywords:** tuberculosis and other mycobacteria, HIV/AIDS and other retroviruses, bacteria, viruses, tuberculosis preventive therapy, antiretroviral therapy, Mozambique

## Abstract

We investigated tuberculosis (TB) diagnoses among persons living with HIV recently initiated on antiretroviral therapy in Mozambique during 2021–2024 (N = 341,844). TB diagnosis rates were lower among those who completed TB preventive therapy (3.1/1,000 person-years) compared with those who had an incomplete course (11.0/1,000 person-years) or did not start (21.6/1,000 person-years).

Tuberculosis (TB) remains the leading cause of illness and death among persons living with HIV (PLHIV) (*1*,*2*). In 2024, among »40.8 million PLHIV globally (*3*), there were »620,000 TB cases and »150,000 TB-related deaths (*4*). For PLHIV, HIV antiretroviral therapy (ART) and TB preventive therapy (TPT) reduce TB incidence and contribute to reduced TB deaths (*5*). TPT should be administered to all PLHIV >12 months of age who have no symptoms of active TB (*2*).

In 2024, there were »2.5 million PLHIV in Mozambique; of those, 86% were on ART (*6*). Among all PLHIV were »29,000 incident TB cases and »5,600 TB-related deaths (*4*). Since 2007, Mozambique has greatly expanded TPT among PLHIV, reaching TPT coverage of 89% by March 2024 (D. Respeito et al., unpub. data, https://doi.org/10.1101/2024.11.25.2431776). During 2007–2023, TPT consisted primarily of a 6-month course of isoniazid (INH); in May 2023, a 3-month course of 12 (1/wk) doses of isoniazid and rifapentine (3HP) was introduced in the southern region.

In this study, we aimed to determine the effect of TPT among PLHIV in Mozambique newly initiating ART by TPT completion status using a national data warehouse. We obtained ethics approval from the Mozambique National Bioethics Committee for Health. This activity was deemed research not involving human subjects by the US Centers for Disease Control and Prevention.

## The Study

We retrospectively studied all PLHIV who initiated ART during 2021–2022, using data from Mozambique’s national ART data warehouse, MozART (Appendix, https://wwwnc.cdc.gov/EID/article/32/3/25-1349-App1.pdf); as of December 2024, the warehouse contained deidentified clinical-encounter data from 620 facilities and 1.6 million PLHIV on ART. We excluded PLHIV with previous TB treatment or TB diagnosis within 90 days of ART initiation; we also excluded those without a documented HIV viral load test within 2 years or ART pickup within 3 months of ART initiation, as a proxy for nonengagement in care.

The outcome of interest was any TB diagnosis within 2 years of ART initiation, as documented in medical records or by enrollment in the TB service ward. The primary independent variable was TPT completion, categorized as did not start TPT, incomplete TPT (<170 days for INH; <80 days for 3HP), and completed TPT (>170 days for INH; >80 days for 3HP). Control variables were age at ART initiation (<15, 15–49, >50 years); sex; facility setting (urban/rural) and region; most recent CD4 count (<200 cells/mm^3^, >200 cells/mm^3^) and World Health Organization (WHO) clinical stage (I–IV) at ART initiation; and most recent HIV viral load result (suppressed, <1,000 copies/mL; unsuppressed, >1,000 copies/mL) before or after TB diagnosis or <24 months on ART and time of TPT initiation (at ART start or delayed).

We used Poisson regression with generalized estimating equations to calculate incidence rate ratios (IRRs) and to account for potential correlation between observations within health facilities. We used IRRs to compare completion categories. We calculated incidence of diagnosed TB as the number of PLHIV with TB diagnosis per total person-years divided by 1,000.

During 2021–2022, a total of 505,098 PLHIV were newly initiated on ART; of those, 28,498 (5.6%) were <15 years of age. We included a total of 341,844 (67.7%) in the analysis; 215,357 (63%) were female and 126,487 (37%) male, median age was 30.0 (interquartile range [IQR] 24–39) years, and 82,418 (24%) received ART in the Southern Region (Table 1). Of the 163,254 PLHIV excluded from the analysis, 120,555 (23.9%) did not have a registered viral load, 28,620 (5.7%) did not have an ART pickup within 30 days of ART initiation, and 14,079 (2.8%) received their diagnosis before initiating ART.

**Table 1:** Characteristics of persons newly diagnosed with HIV in study of TB in persons living with HIV initiating ART, Mozambique, 2021–2024*

Characteristic	No TB diagnosis, n = 338,717	TB diagnosis, n = 3,127	Total, N = 341,844
Age, y, median (IQR)	30 (24–39)	35 (26–43)	30 (24–39)
Age group, y			
<15	17,939 (5.3)	298 (9.5)	18,237 (5.3)
15–49	293,225 (87)	2,352 (75)	295,577 (86)
>50	27,553 (8.1)	477 (15)	28,030 (8.2)
Sex			
F	213,804 (63)	1,553 (50)	215,357 (63)
M	124,913 (37)	1,574 (50)	126,487 (37)
Geographic region†			
Central	153,268 (45)	1,295 (41)	154,563 (45)
Northern	104,067 (31)	796 (25)	104,863 (31)
Southern	81,382 (24)	1,036 (33)	82,418 (24)
Last viral load result‡			
Suppressed	312,197 (92)	2,603 (83)	314,800 (92)
Unsuppressed	26,520 (7.8)	524 (17)	27,044 (7.9)
CD4 result			
<200 cells/mm^3^	8,842 (2.6)	326 (10)	9,168 (2.7)
≥200 cells/mm^3^	38,868 (11)	550 (18)	39,418 (12)
Not tested	291,007 (86)	2,251 (72)	293,258 (86)
TPT initiation and completion			
Complete	265,427 (78)	1,550 (50)	266,977 (78)
Incomplete	67,248 (20)	1,346 (43)	68,594 (20)
Not started	6,042 (1.8)	231 (7.4)	6,273 (1.8)
TPT type			
3HP	17,659 (5.2)	224 (7.2)	17,883 (5.2)
Isoniazid	315,016 (93)	2,672 (85)	317,688 (93)
No TPT	6,042 (1.8)	231 (7.4)	6,273 (1.8)
Started TPT within 30 d of ART initiation			
Delayed TPT start	22,805 (6.7)	327 (10)	23,132 (6.8)
Not started	6,042 (1.8)	231 (7.4)	6,273 (1.8)
TPT at ART start	309,870 (91)	2,569 (82)	312,439 (91)
WHO status at initiation			
Not recorded	5,733 (1.7)	63 (2.0)	5,796 (1.7)
Stage I	275,880 (81)	2,117 (68)	277,997 (81)
Stage II	41,366 (12)	551 (18)	41,917 (12)
Stage III	13,408 (4.0)	345 (11)	13,753 (4.0)
Stage IV	2,330 (0.7)	51 (1.6)	2,381 (0.7)
Health facility setting			
Rural	208,149 (61)	1,749 (56)	209,898 (61)
Urban	130,568 (39)	1,378 (44)	131,946 (39)

*Values are no. (%) except as indicated. ART, antiretroviral therapy; IQR, interquartile range; TPT, TB preventive therapy; WHO, World Health Organization; 3HP, 3-month course of 12 (1/wk) doses of isoniazid and rifapentine. †Mozambique’s 11 provinces are categorized into 3 regions: Central (Sofala, Manica, Tete, Zambezia), Northern (Nampula, Niassa, Cabo Delgado), and Southern (Maputo City, Maputo, Gaza, Inhambane). ‡Suppressed, <1,000 copies/mL; unsuppressed, >1,000 copies/mL.

Of 341,844 PLHIV analyzed, 266,977 (78%) completed TPT, 68,594 (20%) had incomplete TPT, and 6,273 (1.8%) did not start TPT (Table 1). Incidence of diagnosed TB was 5.0/1,000 person-years. Compared with those who completed TPT (3.1/1,000 person-years), diagnosed TB was higher among PLHIV who did not start TPT (21.6/1,000 person-years; IRR 6.9 [95% CI 5.7–8.3]) and those with incomplete TPT (11.0/1,000 person-years; IRR 3.5 [95% CI 3.2–3.8]) (Table 2).

**Table 2:** Incidence of TB among persons living with HIV newly initiated on ART after 2 years of follow-up, Mozambique, 2021–2024*

Characteristic	No. TB positive	Person-years	Incidence (95% CI)	Univariate analysis			Multivariate analysis	
				IRR (95% CI)	p value		IRR (95% CI)	p value
TPT completion status								
Complete	1,550	492,835	3.1 (3.0–3.3)	Referent			Referent	
Incomplete	1,346	122,675	11.0 (10.4–11.6)	3.5 (3.2–3.8)	<0.001		3.83 (3.5–4.2)	<0.001
Not started	231	10,705	21.6 (18.9–24.5)	6.9 (5.7–8.3)	<0.001		5.25 (4.4–6.3)	<0.001
Sex								
F	1,553	393,961	3.9 (3.7–4.1)	Referent			Referent	
M	1,574	232,254	6.8 (6.4–7.1)	1.7 (1.6–1.9)	<0.001		1.56 (1.4–1.7)	<0.001
Viral load result†								
Suppressed	2,603	579,825	4.5 (4.3–4.7)	Referent			Referent	
Unsuppressed	524	46,390	11.3 (10.3–12.3)	2.5 (2.3–2.8)	<0.001		2.28 (2.0–2.5)	<0.001
Age group								
15–49	2,352	541,281	4.3 (4.2–4.5)	Referent			Referent	
>50	477	52,035	9.2 (8.4–10.0)	2.1 (1.9–2.4)	<0.001		1.55 (1.4–1.8)	<0.001
<15	298	32,899	9.1 (8.1–10.1)	2.1 (1.8–2.4)	<0.001		1.76 (1.6–2.0)	<0.001
TPT regimen‡								
Isoniazid	2,672	583,015	4.6 (4.4–4.8)	Referent				
3HP	224	32,495	6.9 (6.0–7.9)	1.5 (1.2–1.8)	<0.001			
No TPT	231	10,705	21.6 (18.9–24.5)	4.7 (3.9–5.6)	<0.001			
Health facility setting								
Rural	1,749	392,126	4.5 (4.3–4.7)	Referent			Referent	
Urban	1,378	234,089	5.9 (5.6–6.2)	1.3 (1.1–1.5)	<0.001		1.21 (1.1–1.4)	<0.001
Region§								
Northern	796	185,636	4.3 (4.0–4.6)	Referent			Referent	
Central	1,295	289,951	4.5 (4.2–4.7)	1.0 (0.9–1.3)	0.67		0.84 (0.7–1.0)	0.02
Southern	1,036	150,628	6.9 (6.5–7.3)	1.6 (1.4–1.9)	<0.001		1.51 (1.3–1.8)	<0.001
Latest CD4 result								
>200 cells/mm^3^	550	72,187	7.6 (7.0–8.3)	Referent			Referent	
<200 cells/mm^3^	326	16,597	19.6 (17.6–21.9)	2.6 (2.2–3.0)	<0.001		2.11 (1.8–2.5)	<0.001
Not tested	2,251	537,431	4.2 (4.0–4.4)	0.5 (0.5–0.6)	<0.001		0.58 (0.5-0.7)	<0.001
WHO staging status at ART start								
Stage I	2,117	509,232	4.2 (4.0–4.3)	Referent			Referent	
Stage II	551	77,186	7.1 (6.6–7.8)	1.7 (1.5–1.9)	<0.001		1.61 (1.5–1.8)	<0.001
Stage III	345	24,948	13.8 (12.4–15.4)	3.3 (3.0–3.8)	<0.001		2.62 (2.3–3.0)	<0.001
Stage IV	51	4,201	12.1 (9.0–16.0)	2.9 (2.3–3.8)	<0.001		1.89 (1.5–2.4)	<0.001
Not recorded	63	10,649	5.9 (4.5–7.6)	1.4 (1.1–1.9)	0.01		1.29 (1.0–1.7)	0.06
Started TPT within 30 d of ART initiation‡								
TPT at ART start	2,569	572,983	4.5 (4.3–4.7)	Referent				
Delayed TPT start	327	42,527	7.7 (6.9–8.9)	1.7 (1.5–1.9)	<0.001			
Not started	231	10,705	21.6 (18.9–24.5)	4.8 (4.0–5.8)	<0.001			

*Incidence is cases/1,000 person-years. IRR, incidence rate ratio, TPT, TB prevention therapy, 3HP, 3-month course of 12 (1/wk) doses of isoniazid and rifapentine. †Suppressed, <1,000 copies/mL; unsuppressed, >1,000 copies/mL. ‡Variable not included in multivariate analysis. §Mozambique’s 11 provinces are categorized into 3 regions: Central (Sofala, Manica, Tete, Zambezia), Northern (Nampula, Niassa, Cabo Delgado), and Southern (Maputo City, Maputo, Gaza, Inhambane).

Median time between ART initiation and TB diagnosis was shortest for PLHIV who did not start TPT (182 [IQR 118–323] days), followed by those with incomplete TPT (216 [IQR 141–408] days) and those who completed TPT (418 IQR 294–560] days). Among those who completed TPT, diagnosed TB was higher in male PLHIV (IRR 1.5 [95% CI 1.4–1.7]), younger and older age groups (<15 years, IRR 1.5 [95% CI 1.4–1.7]; >50 years, IRR 1.7 [95% CI 1.5–2.0]), and in the Southern Region (IRR 1.6 [95% CI 1.3–1.9]) (Table 3). Other risk factors were unsuppressed viral load (IRR 2.3 [95% CI 2.0–2.7]), CD4 count <200 cells/mm^3^ (IRR 1.9 [95% CI 1.5–2.3]), and WHO clinical stage II–IV at ART initiation (stage II, IRR 1.6 [95% CI 1.3–1.8]; stage III, IRR 2.5 [95% CI 2.1–2.9]; stage IV, IRR 1.7 [95% CI 1.1–2.7]).

**Table 3:** Incidence of TB among PLHIV newly initiated on ART who completed TPT, Mozambique, 2021–2024*

Characteristic	TB positive	Person-years	Incidence rate (95% CI)	Univariate analysis			Multivariate analysis	
				IRR (95% CI)	p value		IRR (95% CI)	p value
Sex								
F	803	313,190	2.6 (2.4–2.7)	Referent			Referent	
M	747	179,645	4.2 (3.9–4.5)	1.6 (1.5–1.8)	<0.001		1.54 (1.4–1.7)	<0.001
Viral load result†								
Suppressed	1,316	458,416	2.9 (2.7–3.0)	Referent			Referent	
Unsuppressed	234	34,419	6.8 (6.0–7.7)	2.4 (2–2.7)	<0.001		2.35 (2–2.7)	<0.001
Age group								
15–49	1,197	427,626	2.8 (2.6–3.0)	Referent			Referent	
<15	118	23,783	5.0 (4.1–5.9)	1.8 (1.5–2.2)	<0.001		1.50 (1.2–1.8)	<0.001
≥50	235	41,425	5.7 (5.0–6.4)	2.0 (1.7–2.3)	<0.001		1.72 (1.5–2)	<0.001
TPT regimen								
Isoniazid	1,371	461,601	3.0 (2.8–3.1)	Referent			Referent	
3HP	179	31,234	5.7 (4.9–6.6)	1.9 (1.6–2.3)	<0.001		1.07 (0.9–1.3)	0.54
Health facility setting								
Rural	872	305,777	2.9 (2.7–3)	Referent			Referent	
Urban	678	187,058	3.6 (3.4–3.9)	1.3 (1.1–1.5)	<0.001		1.12 (1–1.3)	0.14
Region‡								
Northern	416	159,095	2.6 (2.4–2.9)	Referent			Referent	
Central	533	205,089	2.6 (2.4–2.8)	1.0 (0.8–1.2)	0.95		1.05 (0.9–1.3)	0.60
Southern	601	128,651	4.7 (4.3–5.1)	1.8 (1.5–2.1)	<0.001		1.58 (1.3–1.9)	<0.001
Latest CD4 result								
≥200 cells/mm3	301	58,421	5.2 (4.6–5.8)	Referent			Referent	
<200 cells/mm3	160	13,622	11.7 (10–13.7)	2.3 (1.9–2.8)	<0.001		1.87 (1.5–2.3)	<0.001
Not Tested	1,089	420,792	2.6 (2.4–2.7)	0.5 (0.4–0.6)	<0.001		0.57 (0.5–0.7)	<0.001
WHO staging status at ART start								
Stage I	1,105	403,014	2.7 (2.6–2.9)	Referent			Referent	
Stage II	268	60,760	4.4 (3.9–5)	1.6 (1.4–1.9)	<0.001		1.55 (1.3–1.8)	<0.001
Stage III	131	18,502	7.1 (5.9–8.4)	2.6 (2.2–3.1)	<0.001		2.46 (2.1–2.9)	<0.001
Stage IV	18	2,993	6.0 (3.6–9.5)	2.2 (1.4–3.5)	<0.001		1.70 (1.1–2.7)	<0.001
Not recorded	28	7,566	3.7 (2.5–5.3)	1.3 (0.9–2)	0.13		1.41 (1–2.1)	0.08
Started TPT within 30 d of ART initiation								
TPT at ART start	1,446	466,832	3.1 (2.9–3.3)	Referent			Referent	
Delayed TPT start	104	26,003	4.0 (3.3–4.8)	1.3 (1.1–1.6)	<0.001		1.02 (0.8–1.2)	0.88

*Incidence is cases/1,000 person-years. IRR, incidence rate ratio, TPT, TB prevention therapy, 3HP, 3-month course of 12 (1/wk) doses of isoniazid and rifapentine. †Suppressed, <1,000 copies/mL; unsuppressed, >1,000 copies/mL. ‡Mozambique’s 11 provinces are categorized into 3 regions: Central (Sofala, Manica, Tete, Zambezia), Northern (Nampula, Niassa, Cabo Delgado), and Southern (Maputo City, Maputo, Gaza, Inhambane).

## Conclusions

In Mozambique, TPT was associated with reduced incidence of TB disease among PLHIV, including among those with incomplete TPT. TPT likely averted TB among PLHIV, potentially saving lives and reducing costs to the health system. Despite that success, certain groups had an elevated risk of developing TB disease, even after TPT completion: men, younger and older PLHIV, and those with poor clinical status at ART initiation (unsuppressed HIV viral load, WHO clinical stages II–IV, and low CD4 count). Our findings are consistent with studies on TB incidence after TPT among PLHIV in sub-Saharan Africa (*7*,*8*). In addition, PLHIV in the Southern Region had elevated incidence, which aligns with higher overall TB incidence rates in the region; it might also reflect regional differences in TB services, including TB case detection (*9*; D. Respeito et al.).

PLHIV who completed TPT had a longer median time to TB diagnosis than did those who did not complete TPT. Our results aligned with similar studies in sub-Saharan Africa and India (*10*–*12*). Because TB diagnosis among those completing TPT occurred »14 months after ART initiation, a second course of TPT after a year could be beneficial, particularly among groups with elevated incidence. However, a recent randomized controlled trial did not find additional benefit from a repeat course of 3HP in Ethiopia, Mozambique, and South Africa (*2*,*13*). There is insufficient evidence for additional courses of TPT for PLHIV who completed TPT without new TB exposures (*2*).

Our findings are strengthened by data from a large national patient cohort containing 85% of PLHIV on ART. However, routine data are prone to quality concerns (i.e., data entry errors, poor completion of clinical tools); continual quality assurance activities and rigorous study exclusion criteria helped mitigate potential data-quality bias. We analyzed available programmatic data, which did not include other variables affecting TB (e.g., underlying conditions, body mass index). Misclassification could have occurred, given reliance on PLHIV self-reporting as newly initiated on ART and data cleaning assumptions. Despite those limitations, our study shows high concordance with other studies analyzing the effectiveness of TPT among PLHIV (*14*) and underscores the value of using a national data warehouse for analyzing programmatic outcomes.

In summary, TPT was associated with reduced TB incidence among PLHIV in Mozambique, including those who had not completed TPT. Improving TPT initiation and completion, along with focusing on men, younger and older PLHIV, and those with poor clinical status at ART initiation, could further reduce TB incidence.

Appendix Additional information about tuberculosis in persons living with HIV initiating antiretroviral therapy after TB preventive therapy, Mozambique, 2021–2024.
