# Supplementary material for: Tuberculosis after TB Preventive Therapy in Persons Living with HIV Recently Initiating Antiretroviral Therapy, Mozambique
Source: Emerg Infect Dis. 2026 Mar;32(3):409–13. doi: 10.3201/eid3203.251349 (PMC13016000; doi:10.3201/eid3203.251349)
Supplement: Appendix — Additional information about tuberculosis in persons living with HIV initiating antiretroviral therapy after TB preventive therapy, Mozambique, 2021–2024. [file 25-1349-Techapp-s1.pdf]

Article DOI: https://doi.org/10.3201/eid3203.251349

EID cannot ensure accessibility for supplementary materials supplied by authors. Readers who have difficulty accessing supplementary content should contact the authors for assistance.

Tuberculosis after TB Preventive Therapy in Persons Living with HIV Recently Initiating Antiretroviral Therapy, Mozambique, 2021–2024

Appendix

Description of the MozART Database

As of November 2025, MozART is primarily an HIV/ART database that contains routine information on patients during their clinical consultations, including screening for TB, TPT initiation and completion, as well as TB diagnosis and treatment. It is derived from the Electronic Patient Tracking System (SESP), which is an Open MRS based system that compiles various clinical forms into a comprehensive health record for PLHIV. Client information is captured on MOH-approved data collection tools or forms, such as the Ficha Mestre (Master Card), Ficha Clinica (Clinical Form), and Ficha Resumo (Summary Form), and entered in SESP manually by data entry clerks. Patient information can also be electronically reported through the interoperability of SESP with the other electronic data systems, including for the pharmacy, laboratory, and community health systems. In addition to the use of MOH-approved tools or forms, a secondary source of patient information is captured through the program enrollment module. Demographic information is captured once in the demographic module and is updated upon entry. MozART systematically integrates patient-level demographic, clinical, and treatment-related data, and centralized data are updated quarterly.

The MozART database is an extraction of retrospectively entered data as captured in the official patient management tools used by clinicians. As of December 2024, MozART is updated with information on over 80% of all PLHIV currently on ART in Mozambique (1.6 million of over 2 million). To mitigate data quality issues, implementing partners complete routine data

validations using a sub-sample of patient charts to check the consistency, completeness, and reliability of data. Additionally, MOH and PEPFAR staff conduct quarterly reviews of the MozART database, validating results against nationally reported figures. While the database itself is not cleaned directly, a thorough cleaning and data transformation process is required before any analysis.

Since MozART contains deidentified data, complete de-duplication is not feasible as there are a portion of patients who decide to silently transfer out of one facility and initiate at another facility. To mitigate this issue, we limited the analysis to only newly initiated on ART and excluded patients that did not have ART pickups within 3 months of their ART start date. Programmatically there have been improvements in the facilitation of patient transfers between health facilities, and the use of a master patient index during registration at the health facility allows clinicians to verify whether a client has been on treatment at another facility before documenting as “new on ART.” For these reasons, we assume that the true level of potential duplication is limited.
